# Analysis of the pathogenicity of novel *GNE* mutations and clinical, pathological, and genetic characteristics of GNE myopathy in Chinese population

**DOI:** 10.1186/s13023-025-03696-2

**Published:** 2025-04-05

**Authors:** Yingming Xing, Lingqian Zhao, Renlong Zhao, Qiyun Liu, Juan Wang, Le Wang, Wei Zhang, Junhong Guo, Rongjuan Zhao, Xueli Chang

**Affiliations:** 1 Department of Neurology, First Hospital, Shanxi Medical University, No.85, Jiefang South Street, Taiyuan, 030012 China; 2 First Clinical Medical College, Shanxi Medical University, Taiyuan, China; 3 Department of Radiology, First Hospital, Shanxi Medical University, Taiyuan, China; 4 Research Center for Neurological Diseases, Shanxi Medical University, Taiyuan, China

**Keywords:** Distal myopathy with rimmed vacuoles, *GNE* compound heterozygous mutation, Promoter mutation, Chinese GNE myopathy

## Abstract

**Background:**

GNE myopathy is a rare autosomal recessive distal myopathy caused by mutations in UDP-N-acetylglucosamine 2-epimerase/N-acetylmannosamine kinase (GNE), a bifunctional enzyme critical for sialic acid biosynthesis. This study aimed to describe a novel autosomal recessive *GNE* pedigree in a Chinese family and explore the possible mechanism of *GNE* variants in GNE myopathy, the most common distal myopathy in China. The clinical, genetic, and pathological characteristics of 216 Chinese patients with GNE myopathy were also summarized.

**Methods:**

The proband and her family underwent a comprehensive medical history assessment and neurological examinations. Whole-exome sequencing was performed on the proband, and Sanger sequencing was performed on family members. 293T cell lines were used for immunofluorescence (IF), Western blot, and dual-luciferase reporter assays. We collected the clinical data of 216 GNE myopathy patients from previous reports up until August 1, 2024. Patients were classified into distinct groups according to mutation location to analyze genotype-phenotype correlation.

**Results:**

Whole-exome sequencing of the proband and Sanger sequencing of all available family members identified a compound heterozygous mutation involving a novel promoter region mutation, c.-259T > C, and a reported mutation, c.88 C > T (p.Q30*). The *GNE* promoter fragment (-500 to -1; c.-259T > C) was cloned to construct the firefly luciferase reporter vector. The dual-luciferase reporter assay showed that the mutated promoter exhibited reduced transcriptional activity, resulting in decreased GNE expression. Western blot and IF analysis of overexpressing Q30* revealed that it reduced GNE expression without altering cellular localization and increased the ectopic cytoplasmic expression of TDP-43. The p.D207V mutation was the most common variant in China. Patients carrying p.D207V tended to experience later disease onset. In the epimerase/epimerase group, men experienced earlier disease onset than women (*p* < 0.05). In other groups, age at disease onset in females was earlier than that in males.

**Conclusions:**

The c.-259T > C mutation decreases promoter activity, while the c.88 C > T (p.Q30*) mutation reduces GNE expression and affects TDP-43 distribution, thus affecting normal cellular function. The p.D207V mutation is the most common *GNE* variant in China and is associated with milder disease progression.

**Supplementary information:**

The online version contains supplementary material available at 10.1186/s13023-025-03696-2.

## Introduction

GNE myopathy, also known as Nonaka myopathy, distal myopathy with rimmed vacuoles (DMRV), or hereditary inclusion body myopathy (HIBM), is a rare, autosomal recessive myopathy typically presenting between the ages of 20 and 40. It is characterized by tibialis anterior muscle weakness [1]. A hallmark of this disease is the involvement of lower limb muscles, progressing from anterior to posterior within the calf, with relative sparing of the quadriceps femoris. Loss of ambulation typically occurs 10 to 20 years after symptom onset. Upper limb involvement may manifest 5 to 10 years after onset, though progression from distal to proximal is not always observed. In later stages, the neck and axial muscles are affected [2]. Current evidence suggests that on histopathological examination, rimmed vacuoles are primarily observed within the muscle fibers [3].

GNE myopathy is indeed caused by bi-allelic mutations in the *GNE* gene, which encodes the UDP-N-acetylglucosamine 2-epimerase/N-acetylmannosamine kinase and is the rate-limiting enzyme for sialic acid biosynthesis [4]. To the best of our knowledge, nearly all reported *GNE* mutations across various ethnic populations around the world are missense or nonsense mutations, while a small number of mutations affecting pre-mRNA splicing have been documented [5, 6].

However, the pathogenicity of gene mutations in the promoter region has rarely been reported. Promoters are now understood to interact with transcription factors that determine the initiation and timing of gene transcription. The promoter, which does not encode a protein, contains the RNA polymerase binding site, the enzyme controlling mRNA synthesis [7]. Promoter mutations are associated with altered transcriptional activity mediated by changes in the binding of trans-acting protein factors to specific DNA sequences within the promoter region. Thus, promoter mutations affect gene expression [8].

This study investigated a case of GNE myopathy resulting from a rare compound heterozygous mutation involving a novel promoter region mutation (c.-259 T > C) and a known mutation (c.88 C > T [p.Q30*]). The promoter region reduced transcriptional activity and GNE expression, while the exon region reduced GNE expression without altering cellular localization and increased ectopic cytoplasmic expression of TDP-43. Clinical, genetic, and pathological characteristics of 216 Chinese GNE myopathy patients were summarized to review the mutation spectrum and genotype-phenotype correlation.

## Materials and methods

### Clinical evaluation

A pedigree associated with GNE myopathy was compiled from patients at the First Hospital of Shanxi Medical University, Taiyuan, China. The study included three participants (one male, two female), with one affected individual and two unaffected individuals. Pedigree analysis suggested autosomal recessive inheritance. Informed consent was obtained from all individuals included in the study. Detailed medical history and physical examination were obtained by a qualified investigator (J.G.). Genomic DNA was extracted from peripheral blood samples (I-1, I-2, and II-1) using standard procedures.

We also searched the PubMed and China National Knowledge Infrastructure (CNKI) databases for previous reports pertaining to Chinese patients with GNE myopathy, totaling 216 patients. The following keywords were used for the database search: “GNE myopathy”, “HIBM”, “DMRV”, and “Nonaka myopathy”. All publications in English or Chinese language were eligible for inclusion. We reviewed the detailed data pertaining to onset age, disease course, muscle weakness, creatine kinase (CK) level, and electromyography (EMG) findings. Individuals with biallelic mutations in the *GNE* gene were included for genotype-phenotype analysis. All persons gave their informed consent prior to their inclusion in the study. This study was reviewed and approved by the Ethics System Committee of the First Hospital of Shanxi Medical University (KYLL-2024-220).

### Whole-exome sequencing and data analysis

Whole-exome capture of subject II-1 was performed using Running Gene (Beijing, China) as described previously. Exome sequencing data was aligned to the human reference genome (hg19) using Illumina Sequence Control Software (SCS) and the BWA Aligner. Single-nucleotide polymorphisms (SNPs) and small insertions/deletions (INDELs) were analyzed with the Genome Analysis Toolkit (GATK) and annotated using ANNOVAR. Clinically significant variants were filtered by: (1) retaining variants with a minor allele frequency (MAF) less than 0.5% in dbSNP, genomAD, and ExAC databases; (2) selecting for exonic non-synonymous SNVs, splice site SNVs, and INDELs; and (3) classifying variants as either disease-related or unrelated.

### Primer construction and sanger sequencing

Clonal primers were constructed for *GNE* mutation sites identified in the proband through whole-exon sequencing. Polymerase chain reaction (PCR) was subsequently performed using blood DNA samples from all available family members. Sanger sequencing was then employed to confirm variant accuracy and perform co-segregation analysis between the gene and disease phenotypes. Primer design was executed using the Primer3 software (http://frodo.wi.mit.edu/). The primer sequences used are detailed in Table S1. A control group comprising 200 unrelated subjects, recruited from Shanxi, China (the region of origin for the affected family), was utilized. All controls exhibited no GNE myopathy-associated phenotypes. Sequence analysis was conducted utilizing the BigDye Terminator Cycle Sequencing Kit. PCR products were evaluated using the ABI PRISM 3730 Analyzer (Applied Biosystems, USA).

### Plasmid construction

Plasmids pGL3-Basic, pGL3-Basic-*GNE* promoter (-500~-1) (pGL3-Basic-WT), pGL3-Basic-*GNE* promoter (-500~-1; c.-259 T > C) (pGL3-Basic-MUT), pRL-TK, pcDNA3.1-EGFP, pcDNA3.1-HA-GNE-T2A-EGFP (GNE-WT) and pcDNA3.1-HA-GNE (c.88 C > T)-T2A-EGFP (GNE-Q30*) were purchased from PPL plasmid and protein sharing libraries (http://www.geneppl.com/). All generated plasmids were validated through Sanger sequencing. The primer sequences are listed in Table S2.

### Cell culture and transfections

293T cells were cultured in Dulbecco’s Modified Eagle Medium (DMEM) (11965092, Thermo Fisher Scientific, Waltham, MA, USA), supplemented with 10% fetal bovine serum (10091, Thermo Fisher Scientific, Waltham, MA, USA) at 37 °C and 5% CO_2_ atmosphere. The cells were transfected with a transfection reagent (101000046, jetPRIME, USA). After 48 h of incubation, protein and total RNA were extracted from the cells.

### Western blot analysis

293T cells were cultured in six-well plates and transfected with jetPRIME transfection reagent (lot no. 101000046, USA). After 48 h, samples were collected, lysed with SDS buffer (P0013G, Beyotime, China), and centrifuged to obtain protein extracts. Protein quantification was performed using the BCA assay. Equal amounts of protein (60 µg) were boiled in sample buffer, subjected to SDS-PAGE, transferred to a PVDF membrane, and immunoblotted with anti-HA antibody (CST3724, Cell Signaling Technology, 1:1000 dilution) and anti-GAPDH antibody (A19056, ABclonal, 1:8000 dilution). Immunoblots were developed using ECL reagent (AR1170, BOSTER, China) and visualized on a ChemiDoc MP imager (Bio-Rad).

### Immunofluorescence assay

293T cells were fixed in PBS containing 4% paraformaldehyde and 0.5% Triton X-100. After 60 min blocking step with 10% fetal bovine serum and 0.5% Triton X-100 in PBS, cells were incubated with primary antibodies: HA-Tag Rabbit (CST3724, Cell Signaling Technology, 1:1000) and anti-TDP-43 (10782-2-AP, Proteintech, 1:500). Subsequently, cells were probed with Alexa Fluor-conjugated secondary antibodies to visualize the subcellular localization of GNE and TDP-43. Nuclei were stained with 4’,6-diamidino-2-phenylindole (DAPI). Images were captured under a confocal microscope (Leica, TSC-SP8).

### Luciferase reporter assay

For the luciferase reporter assay, cells were transfected with the relevant plasmid for three days and washed twice with DPBS. A TransDetect Double-Luciferase Reporter Assay Kit (Transgen Biotech) was used to measure Renilla and Firefly activity according to the manufacturer’s instructions.

### Genotype–phenotype correlation

The 196 patients were categorized into nine groups based on mutation location: E/E (epimerase/epimerase), E/K (epimerase/kinase), E/N (epimerase/null), E/UF (epimerase/unknown function region), K/K (kinase/kinase), K/N (kinase/null), K/UF (kinase/unknown function region), N/N (null/null), and UF/UF (unknown function region/unknown function region). “Null” mutations encompassed frameshifts, nonsense mutations, canonical ±1 or 2 splice sites, multiexon deletions, mobile element insertions, or initiation codon mutations. The age at disease onset was compared across these groups, and additionally, between male and female patients within each group.

### Data presentation and statistical analysis

All experiments were repeated at least three times. Quantitative data were expressed as mean ± standard error of the mean (SEM). Statistical analyses were conducted using GraphPad Prism software (version 6). A Student’s t-test or one-way ANOVA with Dunnett’s post-hoc test was performed to compare means between two or more than two groups, respectively. A *p*-value < 0.05 was considered statistically significant.

## Results

### A GNE myopathy family

**Fig. 1:**
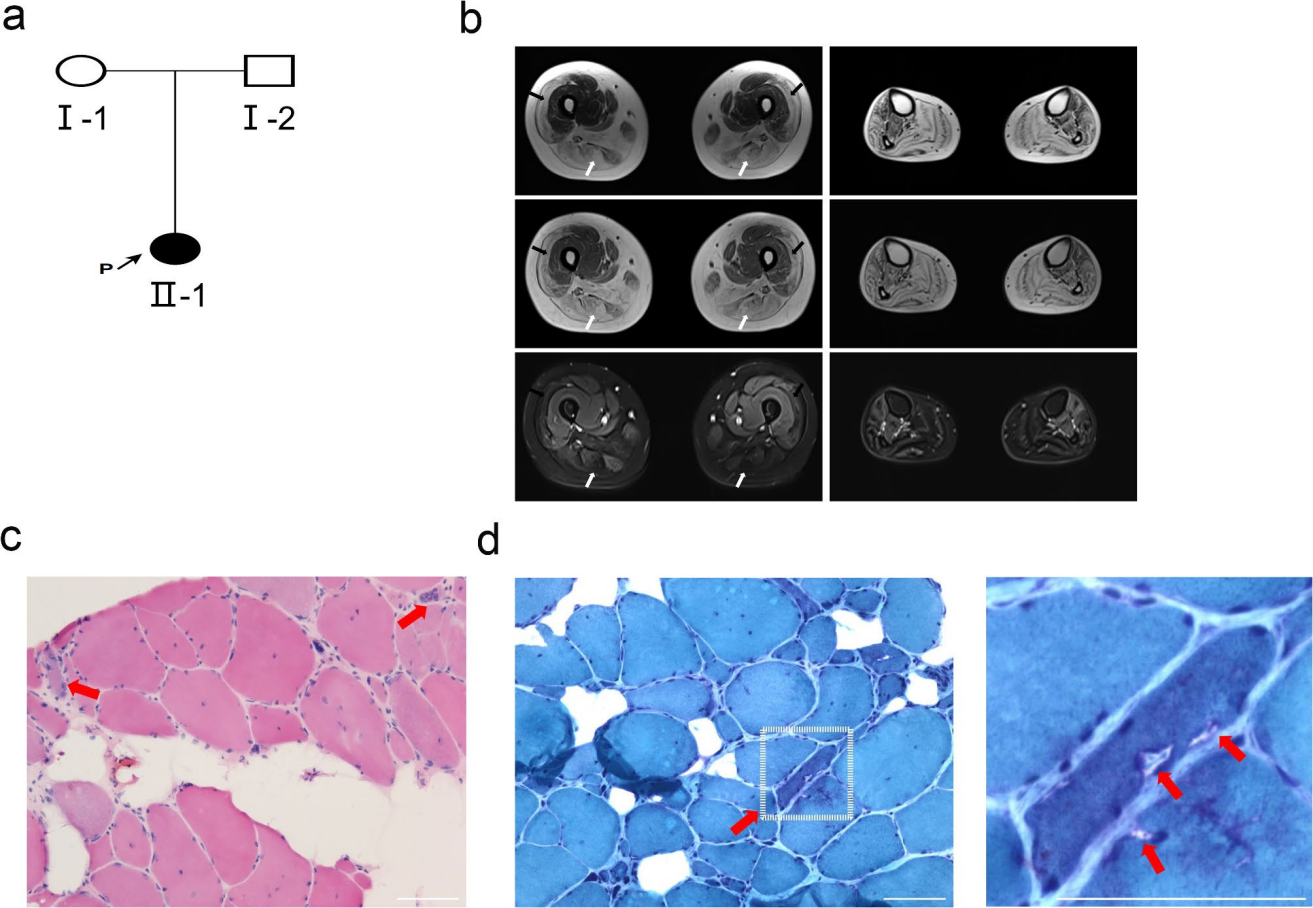
Pedigree of the GNE myopathy along with imaging findings and histopathological features. (**a**) Pedigree of the family exhibiting the *GNE* mutation. Squares denote males, and circles denote females. White and black symbols represent unaffected and affected individuals, respectively. The proband (II-1) is indicated by an arrow. (**b**) Representative T1-weighted, T2-weighted, and STIR-weighted axial MRI scans of the proband’s thighs (left panels, top to bottom) and calves (right panels, top to bottom). Thigh MRI reveals atrophy and fatty infiltration, predominantly affecting the medial muscle groups (white arrows), with involvement of the quadriceps muscles (black arrows). Calf MRI demonstrates edema within the tibialis anterior, tibialis posterior, and extensor digitorum longus muscles, more pronounced on the left side. (**c**) Hematoxylin and eosin staining revealed significant connective tissue hyperplasia, fiber size variation, and the presence of both regenerated and necrotic muscle fibers. (**d**) Modified Gomori trichrome staining demonstrated rimmed vacuoles within muscle fibers (left panel). The boxed region in the left panel is enlarged in the right panel, with red arrows highlighting representative rimmed vacuoles. Scale bars represent 50 μm for all images

Figure 1a presents an overview of the family pedigree. The proband (II-1), a 35-year-old female, presented with progressive weakness in both lower limbs. Her symptoms included an inability to walk on her toes, tremors, gait instability, and frequent tripping (Video S1). Medical Research Council (MRC) scale assessment yielded a grade of 4/5 and diminished deep tendon reflexes in all four limbs. Serum creatine kinase was elevated at 252 IU/L (normal, < 200 IU/L). Electrocardiogram (ECG) and echocardiogram results were normal. Needle electromyography revealed mild myogenic changes in the tibialis muscles. Magnetic resonance imaging (MRI) of the lower limbs revealed edema in the tibialis anterior, tibialis posterior, and extensor digitorum muscles, as well as atrophy and fatty infiltration in both thighs (Fig. 1b). Histopathological analysis of a left vastus lateralis muscle biopsy showed variable fiber size, marked connective tissue hyperplasia, rimmed vacuoles and major histocompatibility complex (MHC) class I positivity (Fig. 1c-d, Fig. S1).

The proband’s mother (I-1) and father (I-2) were asymptomatic, with normal physical examinations and no muscle weakness, rigidity, hyperreflexia, or Babinski sign. Based on clinical features and histopathological findings of the proband, GNE myopathy was suspected.

Whole-exome sequencing was performed on the proband to identify the disease-causing gene. The targeted regions achieved 99.8% coverage at a mean read depth of 104.52×. Analysis focused on variants within genes previously associated with GNE myopathy. Compound heterozygous mutations in GNE were identified in the proband, consisting of a novel promoter region mutation (c.-259T > C) and a known mutation (c.88 C > T [p.Q30*]). These findings confirmed the diagnosis of autosomal recessive GNE myopathy.

### Causal variant identification for the GNE myopathy pedigree

**Fig. 2:**
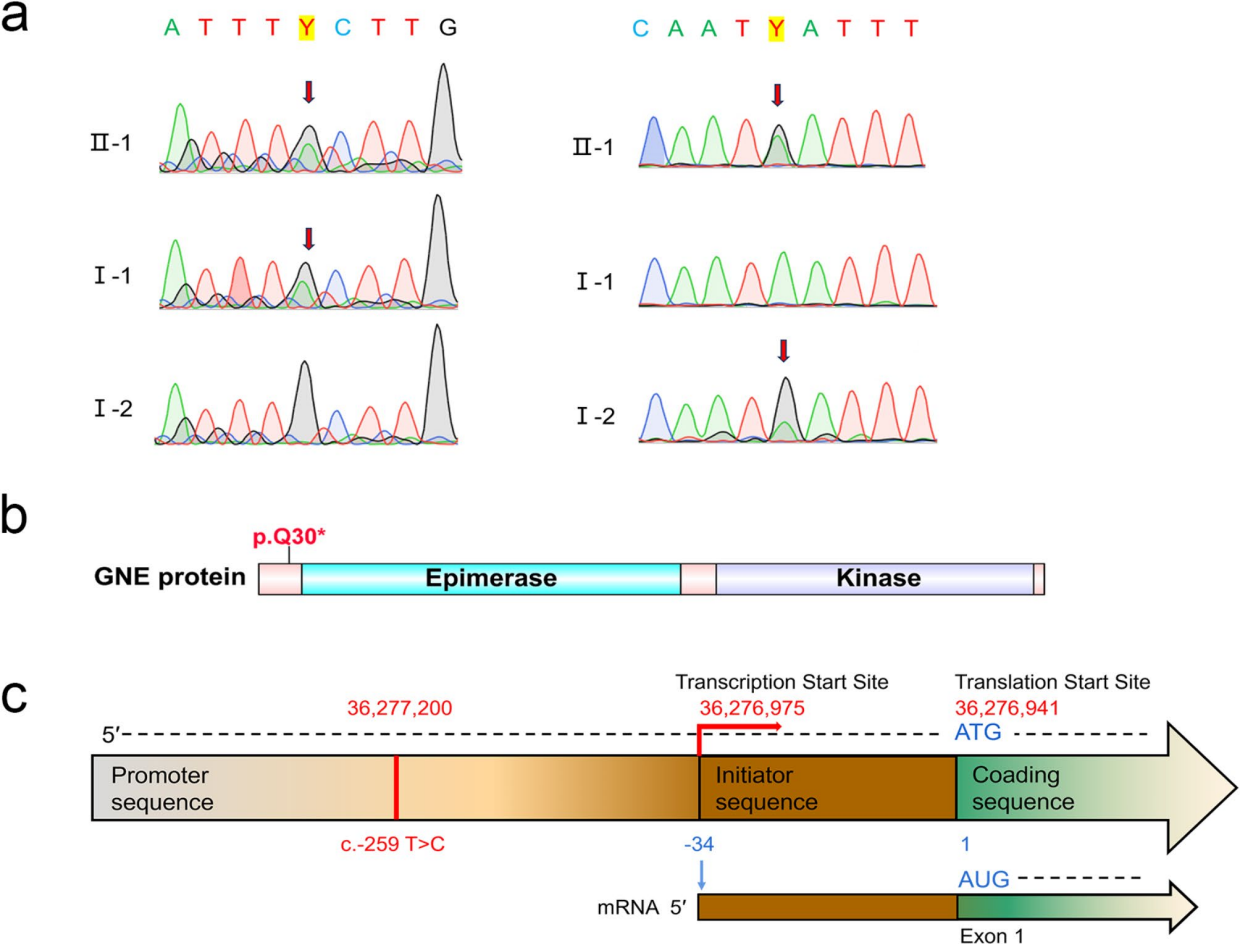
*GNE* variants in the affected family and GNE protein domains (**a**) Partial GNE nucleotide sequences demonstrating the c.88 C > T variant (left) and the c.-259 T > C variant (right) in family members I-1, I-2, and II-1. (**b**) Schematic representation of the GNE protein’s domain organization and basic structure, generated using Illustrator for Biological Sciences (IBS). The observed variants are indicated, with the p.Q30* variant identified in this study highlighted in red. (**c**) Schematic representation of wild-type *GNE* protein structure, with observed variants indicated and the c.-259 T > C variant identified in this study highlighted in red

The c.-259 T > C and c.88 C > T (p. Q30*) variants in GNE were confirmed by Sanger sequencing. Affected family members with GNE myopathy carried both mutations, while unaffected family members carried only one, demonstrating co-segregation of genotype and phenotype (Fig. 2a). Neither variant was detected in 200 ethnically matched, unrelated controls.

The c.88 C > T single nucleotide variant resulted in a premature stop codon (p.Q30*), located upstream of the GNE protein’s Epimerase domain (Fig. 2b). This variant exhibited a high Combined Annotation Dependent Depletion (CADD) score of 35, predicting a deleterious effect, and a MutationTaster score of 1, indicating a disease-causing potential. The c.-259 T > C variant, located upstream of the GNE protein, was predicted to have a regulatory function (Fig. 2c). This variant exhibited a low CADD score of 7.614. Further evaluation, incorporating experimental data, is required to determine the clinical significance of these mutations.

### The c.-259 T > C mutation decreases transcriptional activity, while GNE-Q30* lowers GNE expression without affecting cellular localization and enhances cytoplasmic TDP-43 expression

**Fig. 3:**
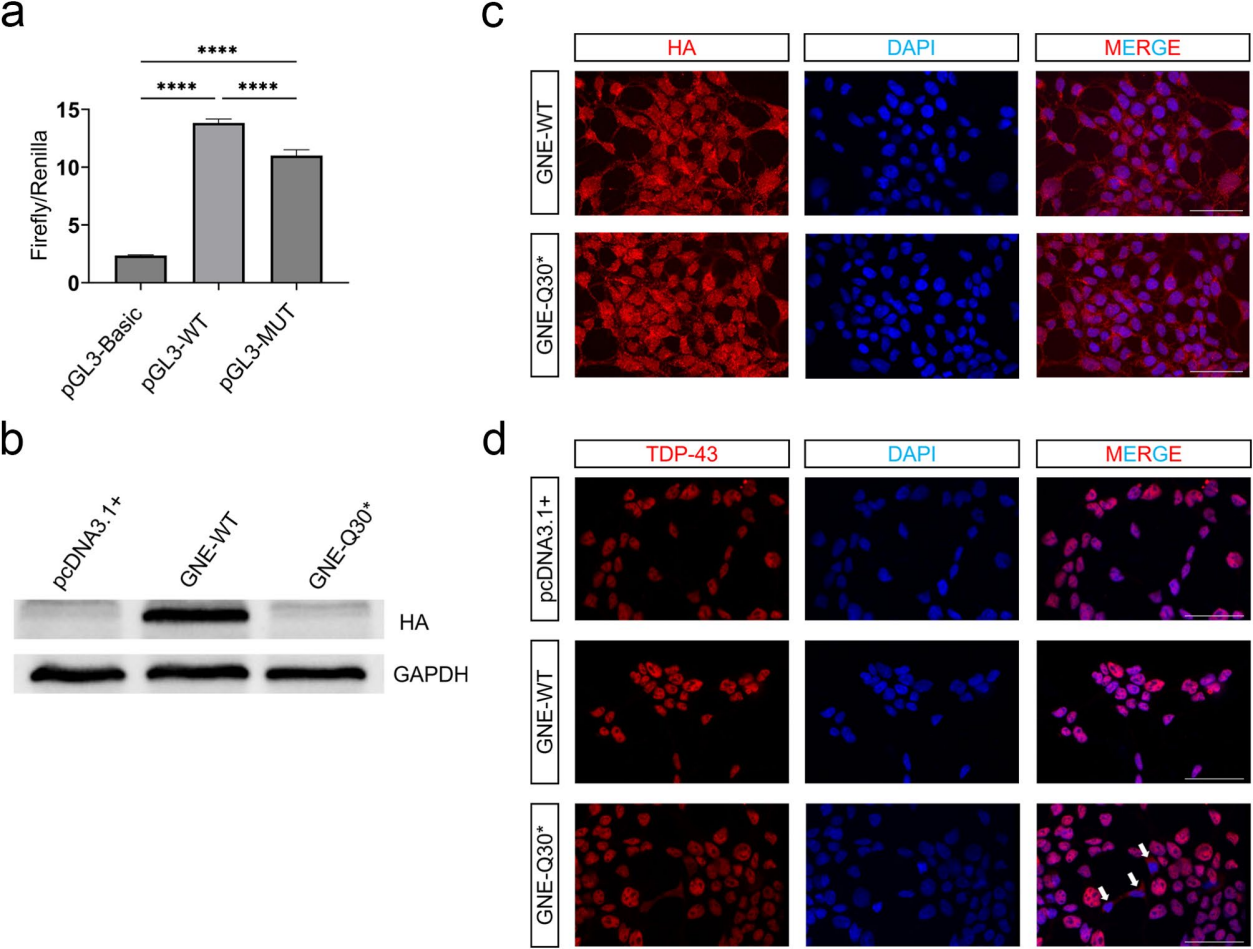
Functional characterization of the c.-259 T > C promoter variant and the GNE-Q30* protein. (**a**) Relative luciferase activity in 293T cells co-transfected with pGL3-Basic, pGL3-Basic-WT (wild-type promoter), pGL3-Basic-MUT (mutant promoter c.-259 T > C), and pRL-TK plasmids. Data are presented as mean ± SEM (*n* = 3 technical replicates). ****p < 0.0001, Student’s t-test (two-sided). (**b**) Western blot analysis of GNE-WT-HA and GNE-Q30*-HA protein expression levels. (**c**) Immunofluorescence analysis of 293T cells transfected with GNE-WT-HA or GNE-Q30*-HA, stained for HA (red) and DAPI (blue). Merged images are shown. (**d**) Immunofluorescence analysis of 293T cells transfected with GNE-WT-HA or GNE-Q30*-HA, stained for TDP-43 (red) and DAPI (blue). Merged images are shown, with white arrows indicating cytoplasmic localization of TDP-43. Scale bars, 50 μm

To analyze the effect on transcriptional activity after promoter mutation, we used a dual luciferase reporter to examine *GNE* promoter activity. We transiently co-transfected pGL3-Basic, pGL3-Basic-WT, and pGL3-Basic-MUT with pRL-TK into 293T cells. Dual-luciferase reporter assays showed that pGL3-Basic-WT and pGL3-Basic-MUT exhibited higher transcriptional activity (*p* < 0.0001) compared to pGL3-Basic (*p* < 0.0001; Fig. 3a). Taken together, these results indicated that the promoter region mutation c.-259 T > C reduced the promoter transcriptional activity resulting in decreased GNE expression.

293T cells were transfected with either GNE-WT or GNE-Q30* to investigate the expression and subcellular localization of the cells. As shown in Fig. 3b, the expression levels of GNE-Q30* were lower than that of GNE-WT (*p* < 0.001). To assess the effect of GNE-Q30* on GNE cell localization, labeling GNE with HA immunofluorescence showed the same localization of GNE-WT and Q30*, both diffuse punctate distributed in the cytoplasm and nucleus (Fig. 3c). These findings collectively suggest that Q30* reduces GNE expression without changing GNE cellular localization.

Next, we sought to determine the expression pattern of TAR DNA Binding protein 43 (TDP-43) in 293T cells transfected with GNE-WT and GNE-Q30*, respectively. TDP-43 is known to be present in the protein aggregates of GNE myopathy and has ectopic sarcoplasmic expression in other myopathies with rimmed vacuoles, including sporadic inclusion body myositis. In 293T cells transfected with GNE-WT, the TDP-43 signal colocalized with nuclear stain, DAPI, as expected. Interestingly, 293T cells transfected with GNE-Q30* exhibited TDP-43 signals in some cytoplasmic regions, with some normal TDP-43 and DAPI overlap (Fig. 3d). These results indicated that GNE-Q30* enabled 293T cells cultured in vitro to exhibit features of GNE myopathy.

### Clinical, genetic, and pathological characterization of GNE myopathy in China

**Fig. 4:**
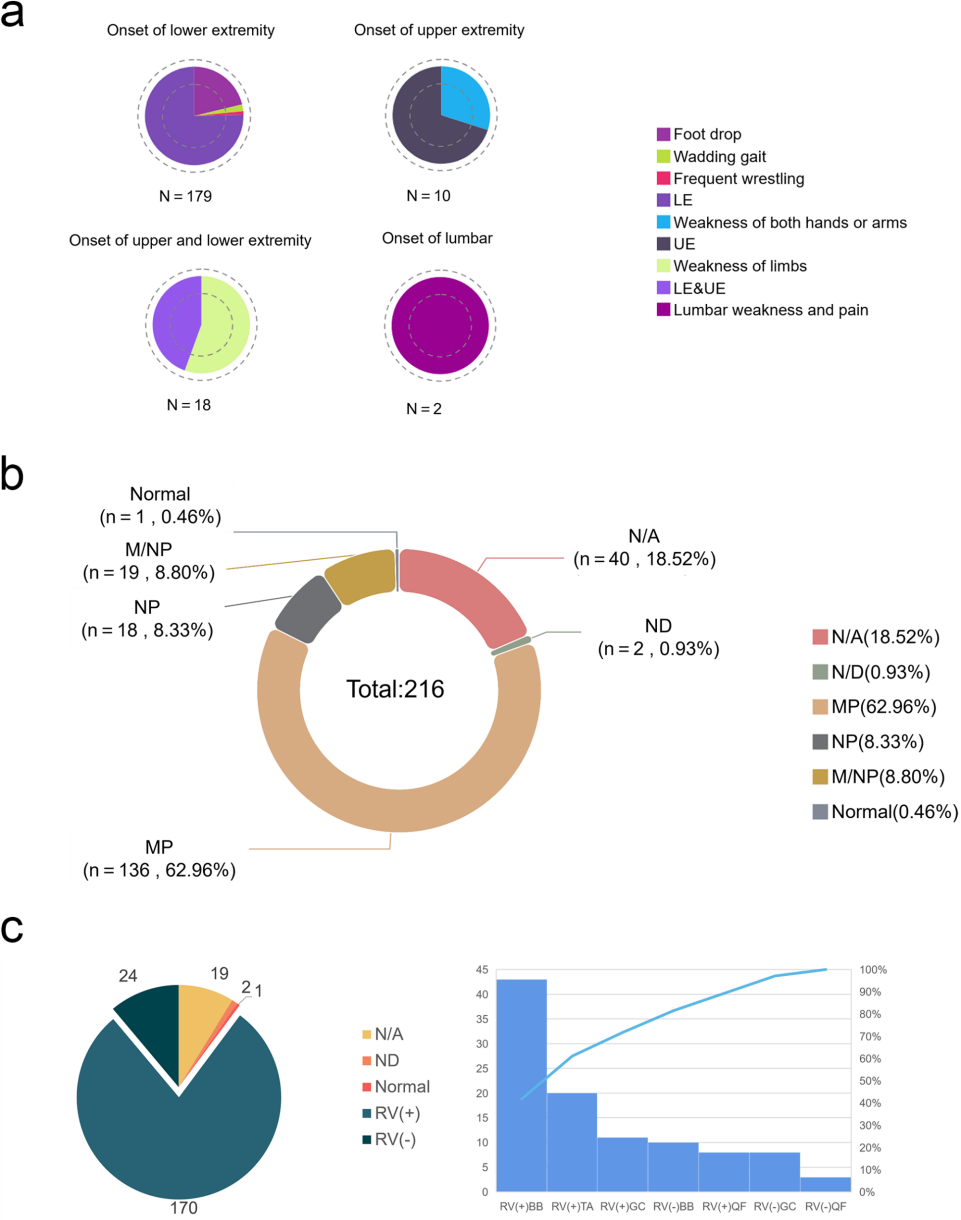
Clinical, electrophysiological, and histopathological findings from 216 patients are summarized. (**a**) Distribution of initial symptom presentation across the cohort, categorized as lower extremity (LE) involvement, or upper and lower extremity (UE&LE) involvement. (**b**) Electrophysiological examination results from the muscle and nerve studies, classified as not available (NA), not done (ND), neuropathic pattern (NP), myopathic pattern (MP), or mixed myopathic and neuropathic pattern (M/NP). (**c**) Muscle histopathology results. The left panel depicts the overall distribution of histopathology results for all 216 patients. The right panel details the specific histopathology findings for the 103 patients with clearly documented biopsy sites, including not available (NA), not done (ND), and rimmed vacuolar (RV) presence

The clinical data and *GNE* variants of 216 patients with GNE myopathy are summarized in Table S3. The cohort comprised predominantly of females (*n* = 124, 57.41%). The mean age at disease onset was 28.06 ±8.15 years (range: 3–60). No significant difference in age at onset was observed between female and male patients (*p* = 0.350). The mean disease duration was 5.99 ± 5.22 years, with no significant difference between female and male patients (*p* = 0.346). While few patients (*n* = 7, 3.24%) did not report initial symptoms, lower limb onset was observed in most patients (*n* = 179, 82.87%), including patients with unilateral/bilateral foot drop (*n* = 38, 21.23%), faltering gait (*n* = 4, 2.23%), and frequent falls (*n* = 2, 1.11%). Upper limb onset was noted in some patients (*n* = 10, 4.63%), including 3 patients (*n* = 3, 30%) with weakness of both hands/arms. Eighteen patients (8.33%) presented with extremity onset, including patients with limb weakness (*n* = 10, 55.56%) and numbness of the extremities (*n* = 1). Two patients (0.93%) presented with lumbar weakness and pain (Fig. 4a). Serum CK levels were measured in mostly all patients (*n* = 203, 93.98%). Elevated CK levels were observed in 167 patients (82.27%), with 7 patients (4.19%) exhibiting CK levels > 2000 U/L. Normal CK levels were observed in 36 patients (17.73%). EMG was performed in most patients (*n* = 174, 80.56%), revealing myogenic changes (*n* = 136, 78.16%), neurogenic changes (*n* = 18, 10.34%), mixed changes (*n* = 19, 10.92%), and normal results (*n* = 1, 0.57%) (Fig. 4b). Eight patients (3.70%) had platelet count data available, of which 3 patients (37.5%) had decreased platelet numbers and subcutaneous bleeding.

Out of the 216 patients, muscle biopsies were performed on 195 (90.28%). Among those biopsied, the biopsy site was clear in 103 (52.82%) patients, while it was unclear in 92 (47.18%) patients. 20.39% (*n* = 21/195) of patients with a clear biopsy site showed absence of rimmed vacuoles with involvement of the quadriceps femoris (*n* = 3, 14.29%), gastrocnemius (*n* = 8, 38.10%), and biceps brachii (*n* = 10, 47.62%). All patients with tibialis anterior biopsies presented with rimmed vacuoles. Additionally, few biopsied patients (*n* = 8, 4.10%) showed rimmed vacuoles in other muscles, indicating that GNE myopathy could also affect the quadriceps femoris (Fig. 4c).

Out of the 216 patients, 15 (6.94%) lacked genetic mutation information, and 5 (2.31%) had information on only one allele mutation. Consequently, the analysis focused predominantly on patients with detailed biallelic mutations (*n* = 196, 90.74%), including homozygous mutations (*n* = 40, 20.40%) and compound heterozygous mutations (*n* = 156, 79.60%). The most common type of mutation observed was missense. The most frequent variant in China was p.D207V (*n* = 54/392, 13.78%) (Table 1). Patients carrying p.D207V exhibited a later age of onset (31.79 ± 9.16) compared to non-carriers (26.88 ± 7.45), which was consistent with previous research (*p* < 0.001).

**Table 1:** Most frequent *GNE* mutations identified in this study

Variants	Amino acid changes	Alleles (%)
NM_001128227.3	NP_001121699.1	This study (*n* = 196*2)
c.620 A > T	p. D207V	54 (13.78)
c.527 A > T	p. D176V	34 (8.67)
c.830G > A	p. R277Q	16 (4.08)
c.1616T > C	p. L539S	11 (2.81)
c.577 C > T	p. R193C	8 (2.04)
c.1523T > C	p. L508S	8 (2.04)
N/A	p. I241S	8 (2.04)
c.649T > C	p. Y217H	7 (1.79)
c.1807G > C	p. V603L	6 (1.53)
c.1571 C > T	p. A524V	6 (1.53)

NM: Reference sequence for mRNA; NP—Reference sequence for protein; nucleotide variants are provided in the mRNA variant 1 (NM_001128227.2) nomenclature and amino-acid substitution are provided in hGNE2 (NP_001121699.1) nomenclature

A cohort of 196 patients was categorized into nine groups based on mutation location and type. The distribution was as follows: E/E (*n* = 74, 37.76%), E/K (*n* = 44, 22.45%), E/N (*n* = 32, 16.33%), E/UF (*n* = 4, 2.04%), K/K (*n* = 30, 15.31%), K/N (*n* = 5, 2.55%), K/UF (*n* = 3, 1.53%), N/N (*n* = 3, 1.53%), and UF/UF (*n* = 1, 0.51%). Mean ages at onset for each group were: E/E (28.22 ± 7.71 years), E/K (29.32 ± 8.99 years), E/N (30.66 ± 9.00 years), E/UF (17.50 ± 6.46 years), K/K (26.10 ± 4.79 years), K/N (24.40 ± 2.61 years), K/UF (22.67 ± 3.06 years), and N/N (25.33 ± 2.52 years). One-way ANOVA indicated a significant difference in mean age of onset across the groups (*p* < 0.05). However, post-hoc analysis using Dunnett’s test did not reveal significant differences between individual groups (Table 2). Within the E/E group, males demonstrated a significantly earlier age of onset compared to females (*p* = 0.025). While not statistically significant, a trend towards earlier onset in females was observed in the E/N, E/K, E/UF, and K/K groups.

**Table 2:** Genotype-phenotype correlation analysis

Genotype	*N*	Age at onset	Sex, F/M	Age at onset of female	Age at onset of male	*p* value
E/E	74	28.22 ± 7.71	46/29	29.80 ± 6.29	25.72 ± 9.06	0.025
E/K	44	29.32 ± 8.99	31/13	27.81 ± 9.48	32.92 ± 6.73	0.085
E/N	32	30.66 ± 9.00	17/15	29.29 ± 6.06	32.20 ± 11.52	0.371
E/UF	4	17.50 ± 6.46	2/2	17.50 ± 6.37	17.50 ± 9.19	1.000
K/K	30	26.10 ± 4.79	18/12	25.44 ± 5.04	27.08 ± 4.40	0.367
K/N	5	24.40 ± 2.61	0/5	-	24.40 ± 2.61	-
K/UF	3	22.67 ± 3.06	1/2	-	23.00 ± 4.24	-
N/N	3	25.33 ± 2.52	0/3	-	25.33 ± 2.52	-
UF/UF	1	-	1/0	-	-	-

*N*, number of affected patients; *F*, female; *M*, male; *E*, epimerase domain; *N*, null, includes frameshifts, nonsense mutations, canonical ± 1 or 2 splice sites, multiexon deletions, mobile element insertion, or initiation codon; *K*, kinase domain; *UF*, unknown function region. *p* value refers to the difference between age at disease onset in female and male patients using the unpaired *t* test

## Discussion

GNE myopathy, a rare autosomal recessive disorder [1], was further characterized in this study through comprehensive clinical evaluation and imaging. While typical clinical manifestations include weakness and atrophy of lower limb muscles, especially the calf muscle, with relative sparing of the quadriceps [9, 10], the proband in this study presented with a variation. MRI of the lower limbs revealed edema in the anterior tibialis, tibialis posterior, and extensor digitorum longus muscles, suggestive of muscle inflammation or injury [11]. Furthermore, atrophy and fatty infiltration were observed in various thigh muscles bilaterally, and histopathological examination of the left lateral vastus muscle showed rimmed vacuoles, further confirming the widespread nature of muscle involvement [1, 2]. These findings diverged to some extent from the existing literature on GNE myopathy. While previous studies have largely focused on calf muscle involvement, this study provides additional evidence of thigh muscle involvement, thereby contributing to a more comprehensive understanding of the clinical spectrum of GNE myopathy.

Prior research has established a link between *GNE* mutations and disease. However, investigations into promoter region mutations remain limited [5, 6]. Promoters are the key regulatory element of gene transcription initiation, and their integrity is essential for gene expression. Garland et al. reported the identification of an 11.3 kb Alu element-mediated deletion mutation in a pair of Indian siblings covering the promoter region of the GNE. This deletion was found to result in a significant reduction in the mRNA and protein levels of the GNE (about 40–50%), indicating that the deletion of the promoter region directly affects the transcription efficiency of the gene [12]. Similarly, a 7.08 kb deletion mutation, also located in the promoter region upstream of the GNE, was identified by Chakravorty et al. RNA sequencing (RNA-seq) showed that the GNE expression was decreased by approximately 50% and only expressed the V727M allele [13]. The present study’s finding of a GNE c.-259 T > C mutation in the promoter region, which affected transcriptional activity and consequently reduced GNE protein expression, aligned with existing literature suggesting that promoter mutations could significantly influence disease development. Using the Human Transcription Factor Database (Human TFDB), we predicted that transcription factors such as BRD4, TCF3, TRIM28, and NHLH1 may bind to a promoter region containing GNE c.-259, which corresponds to transcription factor binding sites. Variations in key nucleotides within these binding sites could potentially alter their binding affinity [8, 14, 15]. Future research should focus on the specific effects of gene promoter mutations on gene expression. Furthermore, investigating how these mutations interact with environmental or other genetic factors and their potential involvement in diverse disease processes constitutes a crucial avenue for future research.

The enzymes encoded by the *GNE* gene are involved in glycosylation and cellular maintenance [16]. TDP-43 is a transcriptional regulatory protein mainly expressed in the nucleus and exhibits abnormal cytoplasmic accumulation in various neurodegenerative diseases [17, 18]. In this study, the GNE-Q30* variant not only resulted in decreased GNE expression but also increased cytoplasmic TDP-43 expression. Previous studies have shown that TDP-43 accumulation is common in myopathies with rimmed vacuoles, suggesting it may represent a common endpoint of muscle cell degeneration rather than a primary pathological mechanism [19]. Therefore, it is widely thought that abnormal TDP-43 accumulation is a feature of GNE myopathy. We hypothesize that this aberrant expression may be related to the cellular stress response as a result of reduced GNE expression, thereby affecting the normal nucleocytoplasmic transport and/or stability of TDP-43 and the reduction of GNE may affect the metabolic status of the cell, which in turn affects TDP-43’s nucleocytoplasmic distribution. This abnormal TDP-43 accumulation may then further disrupt gene expression regulation, exacerbating cellular dysfunction [20–22]. While these findings are consistent with the existing literature on *GNE* mutations and aberrant TDP-43 expression, this study highlights the effect of GNE-Q30* on the distribution of TDP-43 observed in cell culture models, which provides new insights into understanding the molecular mechanisms of GNE myopathy. Future studies are needed to further explore how *GNE* mutations affect TDP-43 nucleocytoplasmic transport and the consequences of these alterations on cellular physiology and pathology. Furthermore, studying the interaction between GNE and TDP-43 and their respective roles in disease progression may facilitate the development of novel therapeutic strategies for GNE myopathy.

The mean age of onset for the E/E, E/K, E/N, E/U, K/K, K/N, K/UF, and N/N groups was analyzed using one-way ANOVA, revealing that the overall mean age of onset varied significantly among the groups (*p* < 0.05). However, post-hoc analysis using the Dunnett test indicated no significant differences between individual groups. This discrepancy might be attributed to the limited sample size in our study, which could have resulted in insufficient statistical power for the analysis. In the E/E group, a statistically significant earlier age of onset was observed in male patients compared to female patients (*p* < 0.05). Although a trend towards earlier onset in female patients was noted in the remaining groups, no significant sex-based differences were observed. Further investigation with a larger sample size is required to confirm these observations. Patients carrying the p.D207V variant exhibited a later symptom onset. Substantial variability in both age of onset and muscle weakness was observed, even among patients sharing the same genetic variant, suggesting the potential influence of additional factors, such as environmental influences, physical activity levels, or other genetic modifiers, on disease progression.

In this study, we confirmed p.D207V as the predominant pathogenic variant in the Chinese population. This aligned with observations reported in prior research. Chen et al. reported a 52.5% incidence of typical rimmed vacuoles in GNE myopathy [23]. Of the 216 patients included in the current study, biopsy site data were available for 103 individuals. Within this subset, typical rimmed vacuoles were observed in 79.61% (*n* = 82/103) of patients. Notably, all patients with tibialis anterior biopsies exhibited rimmed vacuoles. These findings suggest a potential correlation between biopsy site selection and the presence of rimmed vacuoles. However, the limited sample size in the present study, coupled with the reliance on data from published articles, imposes certain limitations on the scope of our analysis.

## Conclusion

This study identified a novel compound heterozygous variant of the *GNE* gene in a proband from a Chinese family, which co-segregated with the observed clinical phenotype within the family. This variant comprises a previously unreported mutation, c.-259 T > C, within a novel promoter region, and a known mutation, c.88 C > T (p.Q30*). Dual luciferase reporter assays demonstrated reduced transcriptional activity of the mutated promoter, resulting in decreased GNE expression. Western blot and immunofluorescence analyses of cells overexpressing the p.Q30* variant revealed diminished GNE expression without alterations in cellular localization and further indicated increased ectopic cytoplasmic expression of TDP-43. Clinical data from 216 Chinese patients with GNE myopathy, constituting the largest such series in this population to date, were compiled and analyzed. This analysis confirmed previous findings that the p.D207V variant is the most common *GNE* variant in China. Moreover, a trend toward earlier disease onset in females than males was observed, with the exception of individuals harboring biallelic mutations within the epimerase domain, where males exhibited earlier onset. The p.D207V mutation was associated with a relatively mild phenotype. This study expands the known genotypic and phenotypic spectrum of GNE myopathy, assesses the pathogenicity of the identified genetic mutations, and provides initial insights into the potential mechanisms by which *GNE* variants contribute to the development of this condition.

## Electronic supplementary material

Below is the link to the electronic supplementary material.

Supplementary Table 1– Primer sequences for PCR. Supplemental Table 2– The primers used in vector construction

Supplemental Table 3– Detailed clinical and pathological features of 216 patients with GNE myopathy

Supplemental Video 1– Video of the proband’s walking posture

Supplementary Fig. 1 Immunohistochemical staining for MHC class I in muscle tissue. Red arrows indicate MHC class I positive skeletal muscle cells, suggesting the presence of an immune-mediated inflammatory response. Scale bars, 50 μm

## Data Availability

Data supporting the findings of this study are available within the paper and its Supplementary Information.
