# Supplementary material for: Analysis of the pathogenicity of novel GNE mutations and clinical, pathological, and genetic characteristics of GNE myopathy in Chinese population
Source: Orphanet J Rare Dis. 2025 Apr 5;20:161. doi: 10.1186/s13023-025-03696-2 (PMC11972457; doi:10.1186/s13023-025-03696-2)
Supplement: Supplementary file 1 — Supplementary Table 1– Primer sequences for PCR. Supplemental Table 2– The primers used in vector construction [file 13023_2025_3696_MOESM1_ESM.docx]

**Table.S1** Primer sequences for PCR

| Mutations | Forward primer (5′ →3′) | Reverse primer (5′ →3′) |
| --- | --- | --- |
| c.88 C>T (p. Q30*) | GAATTTGAAGCTGGGCCCAT | AGTGGTTAAGGACTTGAAACTGA |
| c.-259 T>C | TAGGTTTCCATCCCGAAGCA | AAGCAAGACACGAGCAAGAC |

**Table.S2** The primers used in vector construction

| Name | Forward primer (5′ →3′) | Reverse primer (5′ →3′) |
| --- | --- | --- |
| pGL3-Basic-GNE promoter (-500~-1)-Sanger | CTTTATGTTTTTGGCGTCTTCCA | CTAGCAAAATAGGCTGTCCC |
| pGL3-Basic-GNE promoter (-500~-1; c.-259T>C)-Sanger | CTTTATGTTTTTGGCGTCTTCCA | CTAGCAAAATAGGCTGTCCC |
| pcDNA3.1-HA-GNE-T2A-EGFP-Sanger | CGCAAATGGGCGGTAGGCGTG | CAGGGTCAAGGAAGGCAC |
| pcDNA3.1-HA-GNE(c.88C＞T)-T2A-EGFP-Sanger | CGCAAATGGGCGGTAGGCGTG | CAGGGTCAAGGAAGGCAC |
