# Supplementary figures and images for: Analysis of the pathogenicity of novel GNE mutations and clinical, pathological, and genetic characteristics of GNE myopathy in Chinese population
Source: Orphanet J Rare Dis. 2025 Apr 5;20:161. doi: 10.1186/s13023-025-03696-2 (PMC11972457; doi:10.1186/s13023-025-03696-2)

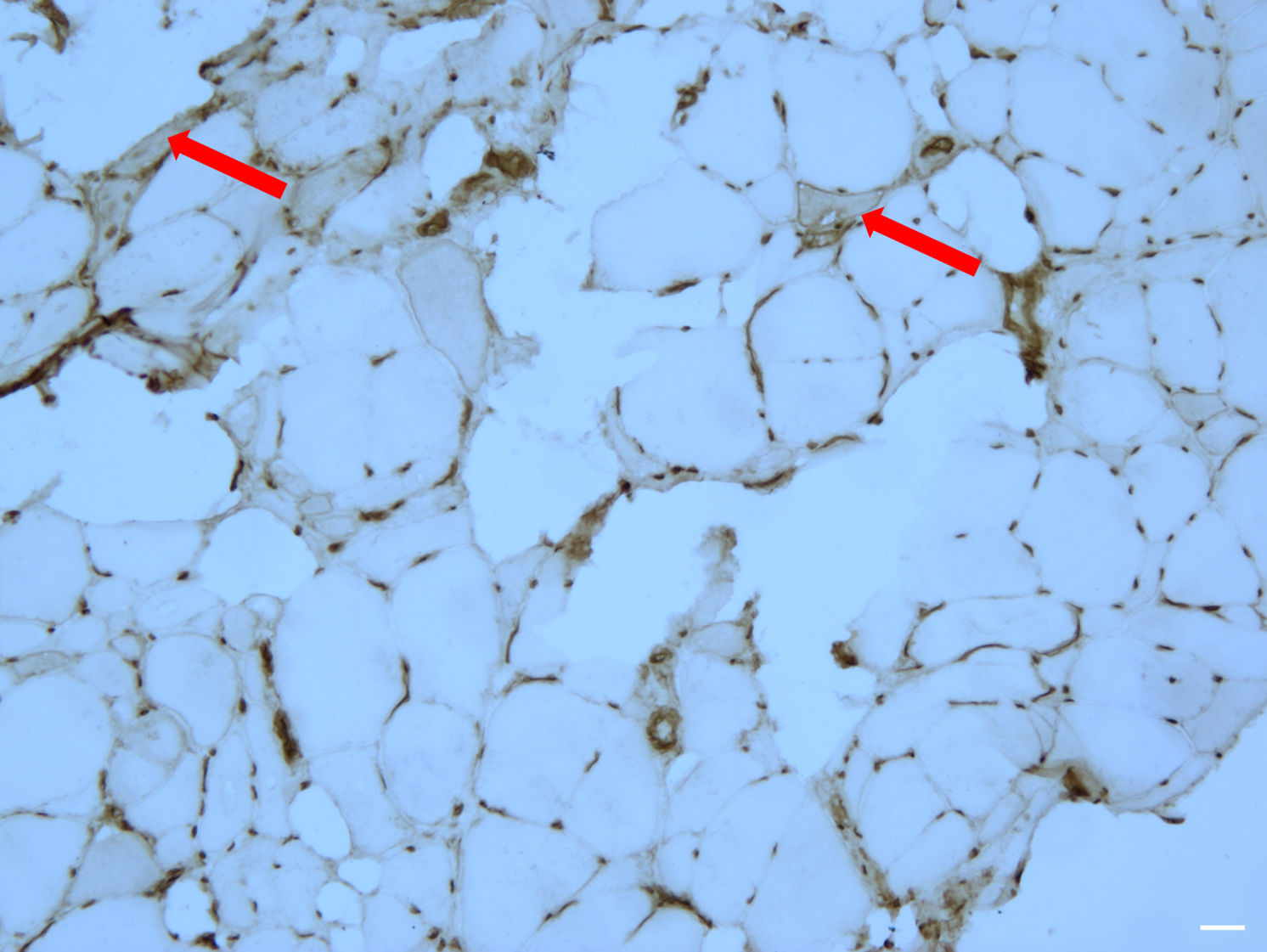

Supplement: Supplementary file 4 — Supplementary Fig. 1 Immunohistochemical staining for MHC class I in muscle tissue. Red arrows indicate MHC class I positive skeletal muscle cells, suggesting the presence of an immune-mediated inflammatory response. Scale bars, 50 μm [file 13023_2025_3696_MOESM4_ESM.tif]
